# Development of an anemia detection model in emergency departments using lip region images based on medical knowledge and deep learning technology

**DOI:** 10.1038/s41598-026-40210-5

**Published:** 2026-06-20

**Authors:** Zhaofan Li, Yugui Zhang, Yuhang Tian, Yizhan Gu, Guanglei Wang, Xin Ning, Qingyue Duan, Jiayi He, Mingyue Zhu, Yunhua Yu, Lili Wang, Li Chen

**Affiliations:** 1 Medical School of Chinese PLA, Beijing, 100853 China; 2 Institute of Semiconductors, Chinese Academy of Sciences, Beijing, 100083 China; 3 Department of General Practice Department, the First Medical Center, Chinese PLA General Hospital, Beijing, 100853 China; 4 Department of of Medical Services, the First Medical Center, Chinese PLA General Hospital, Beijing, 100853 China; 5 Department of Gynecology, Zhujiang Hospital, Southern Medical University, Haizhu District, Guangzhou, 510280 China; 6 Department of Geriatrics, Fuzong Clinical Medical College of Fujian Medical University, 900th Hospital of PLA Joint Logistic Support Force, Fuzhou, 350025 Fujian China

**Keywords:** Anemia detection, Medical knowledge, Deep learning, Object detection, Computational biology and bioinformatics, Diseases, Health care, Mathematics and computing, Medical research

## Abstract

Anemia’s high global prevalence and socio-economic burden necessitate early diagnosis, yet reliance on invasive blood testing creates significant barriers to diagnosis and treatment. To address this, we developed a deep learning model using the Detection Transformer framework for the rapid, non-invasive assessment of anemia severity in a real-world emergency department setting. Comparing a lip-focused model to a full-face approach, the former proved superior, achieving 85.0% accuracy. This significantly outperformed the full-face model (77.0%) and clinical judgments by both senior (59.3%) and junior (49.95%) physicians, with a rapid processing time of 127.50 ms. By integrating key medical knowledge to classify anemia into three severity levels, our model surpasses clinician performance, demonstrating its potential as a powerful, automated tool for clinical decision support.

**Supplementary Information:**

The online version contains supplementary material available at https://doi.org/10.1038/s41598-026-40210-5.

## Introduction

Anemia poses a formidable global health burden, impacting approximately 2 billion people worldwide and resulting in considerable health burdens at the individual level, as well as substantial socio-economic costs^1,2^. Its prevalence is especially pronounced in women of childbearing age (> 30%)^3,4^. This is particularly true in low- and middle-income regions, notably Africa and Southeast Asia, where its consequences are most severe^5^. Furthermore, anemia is a common condition in the geriatric population, affecting an estimated 20.6% of individuals over 60, with even higher rates observed in vulnerable subgroups^6^. Pathophysiologically, anemia is characterized by a reduction in red blood cell mass, resulting in critically low hemoglobin(Hb) levels. As Hb is the principal oxygen transporter, this deficiency directly impairs oxygen delivery to tissues, precipitating a cascade of physiological derangements. The clinical significance of anemia is further highlighted by its strong association with adverse health outcomes, such as cognitive decline^7^, insomnia^8^, diminished physical function^9^, and elevated mortality^10–12^. These associations emphasize the paramount importance of timely diagnosis to facilitate early intervention and improve patient outcomes. Additionally, for patients experiencing clear bleeding, dynamic monitoring of Hb levels is essential to inform clinical decision-making. Despite its importance, the current standard for anemia diagnosis remains invasive blood analysis. This approach is constrained by numerous practical drawbacks, including high resource consumption, the need for skilled technicians, restricted accessibility, and inherent patient discomfort. The cardinal sign of anemia, pallor in the skin and mucous membranes, is a direct visual consequence of reduced Hb concentration. This phenomenon is rooted in the photophysics of Hb: its oxygenated form absorbs blue-green light and reflects red, giving blood its characteristic color. When Hb levels or red blood cell count fall, this red coloration diminishes in regions with dense capillary networks like the cheeks, nail beds, and conjunctiva^13,14^, resulting in a pale appearance. In clinical practice, anemia is often assessed through visual examination of these pallor signs. Although this method of clinical assessment is valuable, its reliability is limited by the inherent challenges in standardization and reproducibility. Moreover, its accuracy remains highly dependent on the clinician’s subjective experience. Therefore, the development of a non-invasive, rapid, accurate, cost-effective, and highly efficient anemia screening tool through the integration of video image processing and detection technologies constitutes a viable and innovative approach.

Grounded in the aforementioned medical knowledge, deep learning-based video images recognition models are capable of detecting and quantifying subtle color variations that are not easily discernible to the human eye. By establishing a mapping relationship between these features and Hb concentrations, the models effectively convert subjective, experience-based evaluations into quantifiable, objective, and verifiable indicators. This advancement facilitates the efficient screening and real-time monitoring of anemia severity.

Although these explorations have yielded preliminary results (Table 1), they have also revealed several critical limitations. First, the classification capability of current models is limited, as most are restricted to binary classification tasks—such as predicting the presence of anemia—thereby impeding enhanced disease management. Second, operational simplicity remains a challenge, as these approaches typically require specialized equipment or direct contact procedures, which significantly constrain their practical application, particularly in resource-limited settings or emergency situations. Moreover, the accessibility of the anemia regression model based on fundus imaging is substantially hindered by the dependency on Optos technology. Third, privacy protection is insufficient. The acquisition of full-face images raises legitimate concerns regarding participant information security, potentially leading to ethical and data security challenges that must not be overlooked.

**Table 1:** Comparison of deep learning models for anemia prediction based on video image data from various body regions.

Author and publication	Task	Model	Input data	Collection equipment	Test (verification)/total data	Performance			Remarks
						AUC	accuracy	MAE(g/dl)	
Mitani^15^	①prediction of anemia ②prediction of moderate anaemia(Hb < 11 g/dl) ③prediction of approximate anaemia(Hb < 12.5 g/dl) ④prediction of Hb(regression model)	Inception-v4	retinal fundus	N/A	10,695/53,473	①0.87 ②0.95 ③0.87	N/A	0.67	
Zhang^16^	①prediction of anemia ②prediction of moderate anaemia(Hb < 9 g/dl) ③prediction of severe anaemia(Hb < 7 g/dl)	InceptionV3	full face	Pad(AIM75-WIFI)	2,098/6,993	①0.84 ②0.69 ③0.82	①82.37% ②68.37% ③74.01%	-	
Zhao^17^	①prediction of anemia ②prediction of Hb(regression model)	InceptionResNetV2	retinal fundus	Optos(Daytona, Optos PLC, Dunfermline, United Kingdom)	1,730/9,221	0.93	N/A	0.83	
Chen^18^	prediction of Hb(regression model)	mobilenetv3 + SEblock	conjunctiva	mobile device( unknown model )	296/1,514	N/A	N/A	1.521	
Mansour^19^	prediction of anemia	Xception	lip	Camera ( unknown model )	414/1,380	N/A	99.28%	-	an adjustable frame was required for acquire only the lip area
Çuvadar^20^	prediction of Hb(regression model)	combined deep learning network	conjunctiva(integrate genders, ages, heights, weights, etc.)	iPhone 6s	total data set: 388 entries	N/A	N/A	0.51	
Kato^21^	prediction of Hb(regression model)	CNN	conjunctiva	iPhone 14	total data set: 150 entries	N/A	N/A	-	the correlation coefficient for the overall predicted data was 0.44
Berghout^22^	prediction of anemia	RexNet	①conjunctiva ②fingernail ③palms	N/A	N/A	①1.0000 ②0.9958 ③0.9918	N/A	-	
Khan^23^	①prediction of anemia ②prediction of Hb(regression model)	InceptionV3	retinal fundus(integrate genders and ages)	VISUCAMlite(Carl Zeiss, Jena, Germany)	899/4,517	0.98	98%	0.58	
Sehar^24^	prediction of anemia	Stacking ensemble model( VGG16、ResNet-50 and InceptionV1)	conjunctiva	iPhone XR	863/4,315	0.97	89.48%	-	
Lin^25^	prediction of Hb(regression model)	BPANet	conjunctiva or palm or fingernails(integrate genders and ages)	iPhone 13(Apple Inc., CA, USA)	54/268	N/A	N/A	2.024	
Navarro^26^	prediction of anemia	DenseNet	nail	Samsung Galaxy A73 5G smartphone	N/A	0.7409	71.08%	-	a specially designed polycarbonate box was required
Fu^27^	prediction of anemia	ResNet50V2 + Conv1D	tongue and Face	the TFDA-1 tongue and face diagnosis instrument	N/A	0.846	81.8%	-	

Grounded in clinical practice and observational evidence, the lip region serves as a critical diagnostic “window” for anemia assessment. This is attributed to its thin stratum corneum, reduced melanin interference^19,27^, dense capillary network^13,14^, and the relative ease of visual inspection and image acquisition. Concurrently, Convolutional Neural Networks (CNNs) have demonstrated immense potential in medical image recognition for assisting disease diagnosis. Their superior performance in automatic feature extraction and correlation construction has driven significant progress in this field^28–32^. Therefore, integrating established medical knowledge with cutting-edge deep learning techniques and building on prior research, this study introduces an image-based analysis of the lip region. This approach offers an effective tool for clinical decision support while incorporating a privacy-preserving design concept. It holds significant potential for rapid triage in emergency departments, large-scale screening in resource-limited settings, and long-term home monitoring with early warning capabilities.

## Results

### Dataset description

**Fig. 1:**
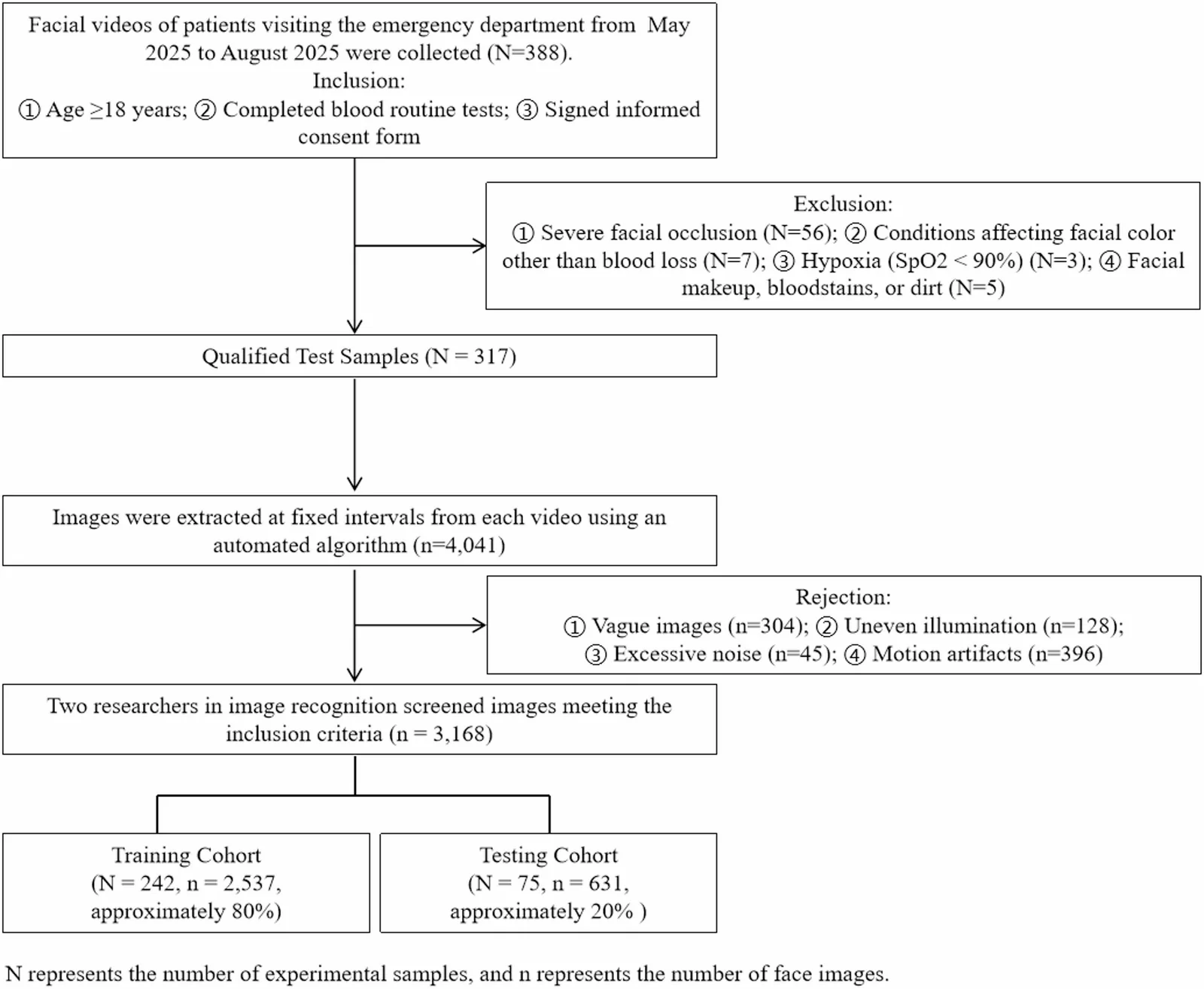
Flowchart of data processing. *Note*: N represents the number of samples, and n represents the number of facial images.

A total of 388 patients were recruited from the Emergency Department of the First Medical Center of the Chinese People’s Liberation Army General Hospital (PLAGH) from May 2025 to August 2025. After applying the exclusion criteria and data processing strategies, 317 patients were ultimately included, yielding a final dataset of 317 facial videos and 3168 facial images suitable for model development. This dataset was randomly partitioned into a training cohort (242 samples, 2537 images) and a testing cohort (75 samples, 631 images). A detailed flowchart of the data acquisition and patient selection process is presented in Fig. 1. For analysis, patients were stratified by Hb level into three groups: Category I (Hb > 9 g/dL), Category II (6 g/dL ≤ Hb ≤ 9 g/dL), and Category III (Hb < 6 g/dL)^33^. The categories were well-balanced, with no statistically significant differences in age or gender (Table 2). Further baseline characteristics of the training and testing cohorts are detailed in Supplementary Material 1.

**Table 2:** Baseline characteristics of the research subjects.

Characteristics	Category I (N = 99,n = 939)	Category II (N = 190,n = 1562)	Category III (N = 28,n = 667)	P value
Age(yd)	66.47 ± 16.96	67(53,76)	62.14 ± 18.43	0.526
Gender,n(%)				0.125
Male	67(67.70)	107(56.30)	19(67.90)	
Female	32(32.30)	83(43.70)	9(32.10)	
Major diseases,n(%)				< 0.01
digestive system	22(22.20)	87(45.80)	12(42.90)	
Circulatory system	28(28.30)	43(22.60)	5(17.90)	
Respiratory system	20(20.2)	16(8.4)	1(3.6)	
Urinary system	3(3.0)	14(7.4)	3(10.7)	
Nervous system	14(14.1)	5(2.6)	1(3.6)	
Musculoskeletal system	8(8.1)	9(4.7)	0(0)	
Hematologic system	1(1.0)	9(4.7)	5(17.9)	
Other body systems	3(3.0)	7(3.7)	1(3.6)	

N represents the number of samples, and n represents the number of facial images.

## Test results

Model A, which focused on lip region image analysis, demonstrated superior overall performance. As presented in Table 3, the model achieved a mean average precision of 82.4%, an average accuracy of 85.0%, and an average recall of 82.0%. The average time required for image loading and inference per case is 127.50 ms, with the average inference time alone being as low as 55.00 ms. Among the three subgroups, Category III achieved the highest prediction accuracy, reaching 86.8%. In comparison, Model B, which focused on comprehensive facial image analysis, achieved a mean average precision of 72.3%, an average accuracy of 77.0%, and an average recall of 71.5%. The average time required for image loading and inference per case is 128.90 ms, with the average inference time alone at 55.30 ms. Among the three subgroups, Category III also achieved the highest prediction accuracy of 79.90%.

**Table 3:** Model performance comparison.

Item	Mean average precision				Average accuracy (%)	Average recall (%)	Average evaluation time (ms)	Average inference time (ms)
	Overall (%)	Category I (%)	Category II (%)	Category III (%)				
Model A	82.4	74.5	86.0	86.8	85.0	82.0	127.5	55.0
Model B	72.3	65.5	71.6	79.9	77.0	71.5	128.9	55.3

**Fig. 2:**
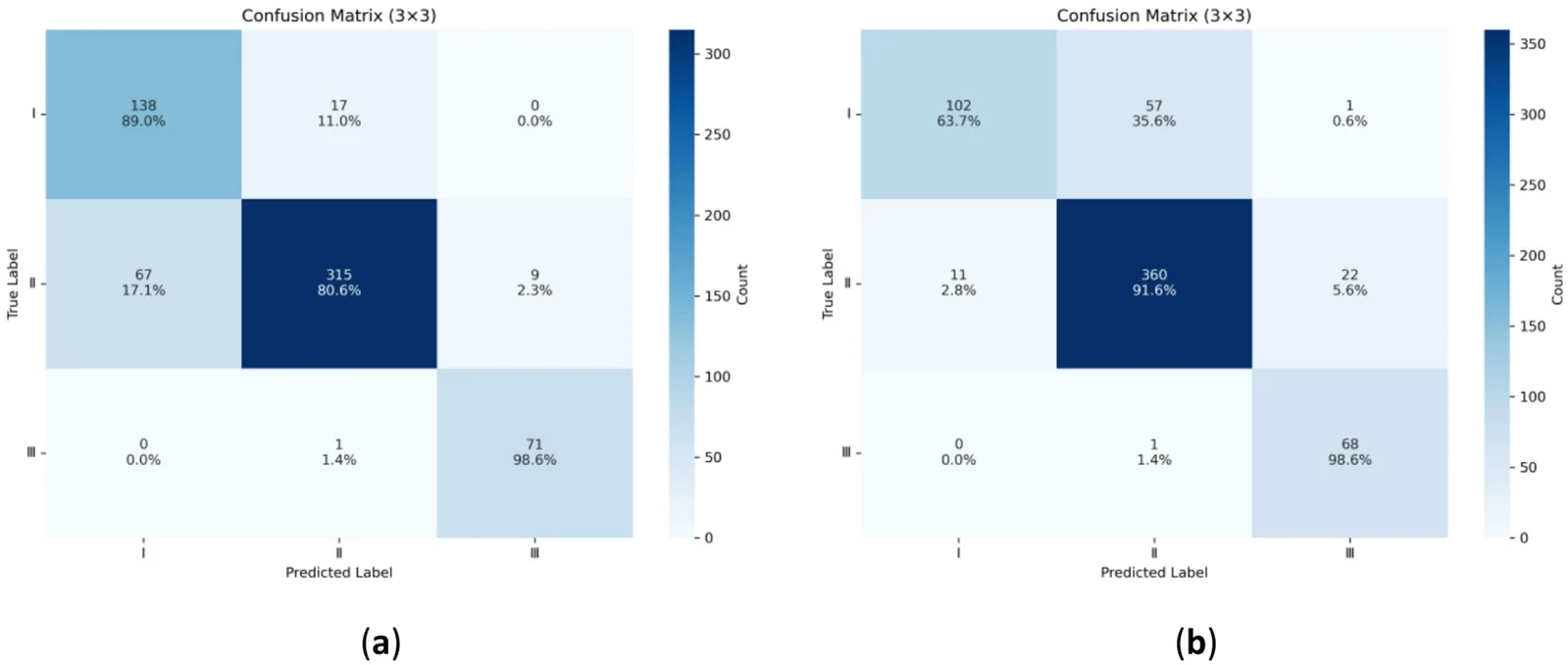
Confusion matrices of each model on the testing cohort. (**a**) Confusion matrices of each model A on the testing cohort; (**b**) Confusion matrices of each model B on the testing cohort.

The classification performance of Models A and B was evaluated via confusion matrices (Figs. 2a, b). Model A demonstrated robust and balanced performance across the test cohort, achieving sensitivities of 89.0% and 80.6% for Category I and II images, respectively. Its performance was exceptional for Category III images (severe anemia), attaining a sensitivity of 98.6% with only a single false negative, which highlights its effectiveness in identifying critical cases. In contrast, Model B was less sensitive than Model A for Category I images but significantly outperformed it in detecting Category II images, suggesting a particular strength in recognizing features of moderate anemia. Nevertheless, both models achieved equally high sensitivities for Category III images (98.6%), highlighting their shared capability in detecting the most severe cases. The reduction in total classified instances observed in the confusion matrices is a direct result of the implemented rejection-based classification strategy. This strategy excludes predictions for samples with low confidence scores (< 0.3). Grounded in established machine learning principles and supported by prior research^34–36^, this approach is especially critical for high-stakes medical applications. By referring low-confidence predictions to experts for review instead of rendering automated decisions, the system significantly enhances diagnostic reliability and accuracy. Crucially, this design mitigates the risk of misdiagnosis associated with uncertain cases, reflecting a fundamental medical safety principle: when in doubt, it is better to abstain from a diagnosis than to risk an error.

The mAP metric and the confusion matrix provided a complementary and robust framework for performance evaluation. Within this framework, Model A significantly surpassed Model B, an advantage stemming mainly from its specialized focus on the lip region, which in turn drove a substantial improvement in overall accuracy.

**Fig. 3:**
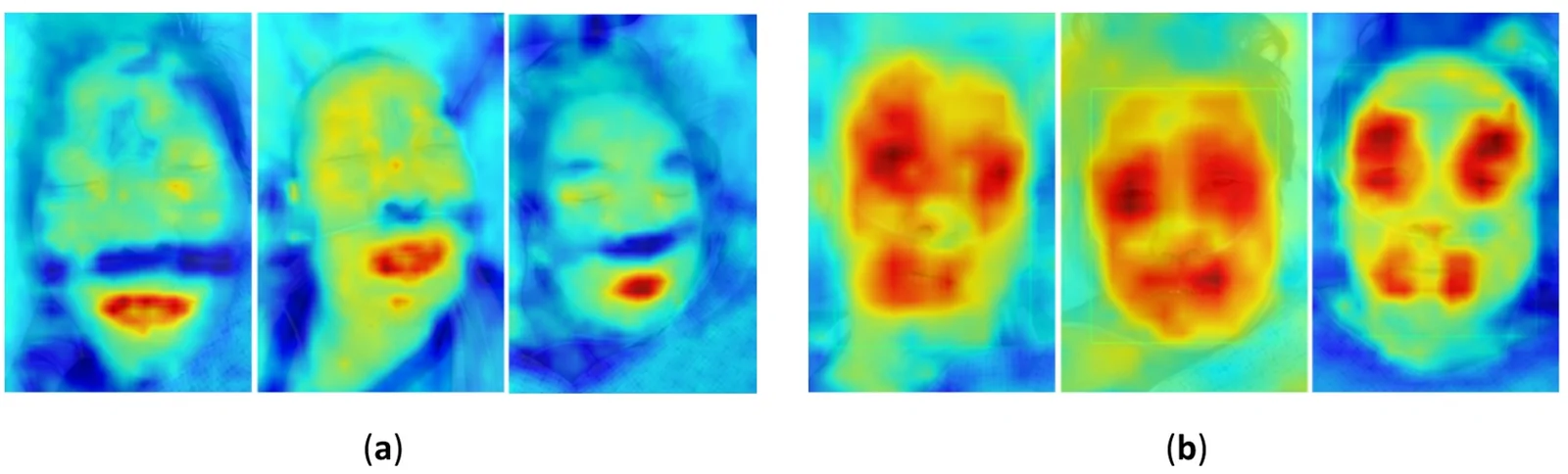
Heatmap visualization. (**a**) Heatmaps of Model A for three samples in the testing cohort; (**b**) Heatmaps of Model B for three samples in the testing cohort.

To visually interpret the model’s decision-making process, we generated heat maps in which color intensity corresponded to the relative contribution of different regions. The heat map for Model A (Fig. 3a) illustrated a highly focused attention pattern, identifying the lip red region as the primary contributor of its predictive output. This finding was in direct alignment with the hypothesis of our experimental protocol. In contrast, the heat map for Model B (Fig. 3b) showed a more distributed attention pattern, with the lips, cheeks, and eyes collectively emerging as key contributing regions. This result not only corroborates findings from prior studies but also aligns with established medical knowledge.

## Physicians interpretation results and comparison

To benchmark the model against human performance, anemia severity for 75 samples in the testing cohort was independently evaluated by two senior physicians (> 8 years’ experience) and two junior emergency practitioners (< 3 years’ experience). The senior physicians achieved an average accuracy of 59.3% with an average evaluation time of 4.62 s per case, outperforming the junior group. Detailed results are presented in Table 4. Model A achieved an evaluation accuracy of 84.00%, which was significantly superior to the physicians’ interpretations (see Supplementary Material 2). Statistical analysis was performed on 74 valid samples (one case was excluded due to missing data). There were 28 pairs of discordant judgments between Model A and the most accurate senior physician. Specifically, the number of samples where Model A was correct but the physician was incorrect (b = 23) was significantly higher than the number where the physician was correct but Model A was incorrect (c = 5). According to the McNemar test principle, the chi-square value was calculated as (b − c)^2^/(b + c) = 11.57. This value is far greater than the critical value of 3.84 for *p* < 0.05. This indicates that, based on the analysis of these discordant pairs, we have sufficient statistical power to reject the null hypothesis. This confirms that the sample size is sufficient and the difference is significant, verifying that Model A’s diagnostic performance is significantly superior to that of the physician.

**Table 4:** Judgment outcomes of senior and junior physicians based on their professional experience.

Evaluation metrics	Average time	Average accuracy (%)	Sensitivity			Precision		
			**Category I** (%)	**Category II** (%)	**Category III** (%)	**Category I** (%)	**Category II** (%)	**Category III** (%)
Senior group	4.62 s	59.30	–	–	–	–	–	–
Senior physician 1	4.16 s	57.30	57.69	58.70	33.33	51.72	67.50	16.67
Senior physician 2	5.07 s	61.30	57.69	65.22	33.33	71.43	73.17	7.69
Junior group	6.56 s	49.95	–	–	–	–	–	–
Junior physician 1	5.75 s	49.30	61.54	47.83	100	64.00	78.57	13.64
Junior physician 2	7.36 s	50.60	34.62	58.70	66.67	100	61.36	9.09

## Discussion

Anemia is a significant global public health challenge, where early diagnosis can substantially reduce healthcare costs. However, conventional diagnostic methods have notable limitations. To address this, our study integrated medical domain knowledge with deep learning to develop a novel anemia detection model that concentrated on lip region imagery. This approach focused on the lip area—which has a strong physiological correlation with anemia severity—instead of indiscriminately extracting features from full-face images. This targeted strategy enhanced detection performance. Additionally, it also offered advantages in terms of convenience and interpretability and held potential for improving privacy protection strategies in facial recognition systems. Built on the Detection Transformer(DETR) architecture, the model achieved a mean average precision of 82.4% and an average accuracy of 85% with a rapid inference time of merely 55.00 ms per case. A comprehensive comparative analysis showed that our model significantly outperformed both full-face models and senior emergency physicians. Compared to full-face models, it demonstrated superior precision (82.4% vs. 72.3%), superior accuracy (85.0% vs. 77.0%) and a slightly faster inference time (55.0 ms vs. 55.3 ms). More importantly, it surpassed senior physicians in both accuracy (85.0% vs. 59.3%) and efficiency (evaluation time: 55.0 ms vs. 4.62 s per case). These results underscored the model’s substantial potential for practical clinical deployment.

In this study, annotated lip region images were selected as the input for anemia detection. This choice is grounded in the following medical knowledge and experiential analysis. The lip region serves as an ideal “window” for anemia detection. Compared to nail beds and palms, the lip region offers superior timeliness and stability. As body extremities, nail beds and palms are the last sites to respond to physiological changes. Moreover, they are susceptible to external factors such as poor perfusion, trauma, infection, and nail polish application^37^. Although the eyelid is also a mucosal region, it presents issues including the risk of cross-infection during eversion, subject intolerance, excessive retraction, and interference from conjunctival secretions^21,25^. Additionally, fundus imaging requires specialized equipment, resulting in low operability and accessibility. Compared to the tongue, the lip region requires minimal subject cooperation and is applicable even to comatose patients. Relative to the cheeks and forehead, the lips are rich in capillaries and possess a thinner stratum corneum, making them more sensitive to hemoglobin level changes. In contrast to full-face images, the lip region reflects the anemic state with greater focus. This reduces interference from irrelevant features during model judgment. Comparative experiments confirmed this; despite utilizing more information, the full-face model demonstrated degraded performance. Therefore, precise localization of the region of interest (ROI) is crucial to prevent the model from learning incorrect patterns.

This study extends beyond binary classification to implement a three-class model for anemia detection. Inspired by Akmal^38^, who developed a three-class prediction model for fetal conditions (normal, suspected, pathological), this approach aims to enhance the refined and process-oriented management of anemia patients. Based on clinical significance, we adopted widely recognized standards for anemia severity to define the three categories^33^. Category I (Hb > 9 g/dL) includes non-anemic and mildly anemic patients. With relatively mild symptoms and risks, clinical intervention primarily involves nutritional support alongside treatment of the underlying condition. Category II (6 g/dL ≤ Hb ≤ 9 g/dL) corresponds to moderate anemia. Timely blood transfusion intervention must be considered based on factors such as the underlying condition and age. Category III (Hb < 6 g/dL) encompasses severe and very severe anemia, necessitating comprehensive treatment measures and close monitoring. Integrating this refined classification with rapid assessment guides clinical decision-making by tracking changes in anemia severity. This provides critical support for early risk warning in emergency or rescue settings. Mitani^15^ also emphasized that, in the context of potential disease progression, the change in Hb levels across visits is clinically significant—not just the Hb value at a single visit.

This study employed the DETR as its foundational architecture, a choice that conferred significant advantages over conventional Convolutional Neural Networks(CNNs). Firstly, DETR’s self-attention mechanism enables the simultaneous extraction of both global contextual information and fine-grained local visual features^39^. This capability is critical for accurately assessing anemia severity, as identifying subtle chromatic variations—from a healthy redness to the pallor indicative of anemia—requires focused analysis of localized pixel intensities in the lip area, combined with a comprehensive comparative evaluation of lip color relative to adjacent regions. Secondly, the DETR model, pre-trained on a diverse dataset, was equipped with extensive color-related features^40^. Through transfer learning, this inherent color sensitivity was not only preserved but also refined, enhancing the model’s ability to identify subtle lip color variations and improving the overall efficiency and precision of feature extraction. Moreover, the integrated ResNet-50 backbone excelled at capturing critical low-level features like color and texture^41^, which were essential for constructing high-resolution feature maps. These maps were then supplied to the upper-layer Transformer, enabling the accurate detection of minute color differences in the lip area. Thirdly, the Transformer architecture offers inherent adaptability to spatial transformations, allowing the model to effectively process images from various angles and thereby enhancing its robustness in real-world scenarios ^42^. Finally, in contrast to the multi-stage pipelines of traditional CNNsDETR facilitates a direct mapping from image input to anemia grade. This end-to-end approach not only streamlines model design but also minimizes the cumulative errors inherent in multi-stage processing.

The paradigm for anemia detection has undergone a significant shift from invasive, contact-based procedures to non-invasive, non-contact solutions, greatly enhancing patient convenience and tool accessibility. This Progress has spurred considerable research efforts. A notable example is the work by Khalafallah et al.^43^, who leveraged Rainbow signal extraction technology with a Masimo Pronon-7 multi-wavelength (500–1300 nm) pulse oximeter to enable spectrophotometric pulse hemoglobin (SpHb) monitoring via spectrophotometry. This method demonstrated performance comparable to conventional laboratory tests. Similarly, Mehmood et al.^44^ developed an optical wristband that estimates Hb levels by measuring light transmittance through the finger. Grounded in the Beer-Lambert law, this approach facilitates continuous, non-invasive monitoring. Despite these promising advances, the promotion and application of such devices faces substantial barriers. For instance, high manufacturing costs and susceptibility to environmental interference continue to hinder their real-world implementation. The advent of artificial intelligence, particularly deep learning, is catalyzing a paradigm shift in medical diagnostics. A growing body of evidence demonstrates that patient-specific biometric features—including voiceprints, gait patterns, and facial features—serve as powerful biomarkers that can significantly enhance diagnostic accuracy. This is exemplified by depression, where patients often exhibit prolonged verbal response times. Leveraging this insight, Yamamoto et al.^45^ utilized automatic speech recognition to investigate the strong correlation between specific speech features (such as speech rate, pause duration, and response latency) and the severity of depressive symptoms. Similarly, freezing of gait (FOG), a prevalent motor symptom of Parkinson’s disease characterized by difficulty in lifting the feet and initiating movement—often with lower limb tremors—has also been a target for AI analysis. Building on these clinical characteristics, Li et al.^46^ extracted motion-related features from walking videos to develop a machine learning-based FOG recognition model. This model achieved an accuracy of 81.56%, a sensitivity of 87.5%, and a specificity of 79.82%, showcasing its potential for community-based Parkinson’s screening. Indeed, while research on various biometric markers has proven valuable, the extraction and analysis of facial features stands out as the most prominent in this domain. This potential is powerfully articulated by Boyd et al.^47^, who posited that facial features can provide important clues about a patient’s condition. Clinically, several characteristic facies are well-established indicators. These include the hepatofacial facies, presenting with a grayish-black discoloration of the face; the icteric facies, defined by jaundice of the skin and sclera; the nephrotic facies, which manifests as periorbital edema; and the distinctive cherry-red lips associated with carbon monoxide poisoning. Building upon this established medical knowledge, the integration of advanced deep learning methodologies has enabled the development of facial recognition-based predictive models. Notably, a facial feature-driven model for hyperbilirubinemia prediction has demonstrated a sensitivity of 80.0% and a specificity of 92.6%, employing a serum bilirubin threshold of 1.5 mg/dL as the cutoff value^48^. Furthermore, a facial recognition system designed to estimate blood oxygen saturation (SpO2) has achieved an average absolute error of ≤ 2%^49^. Taken together, these findings underscore the significant potential of intelligent facial recognition systems for non-invasive, early-stage disease detection and health status assessment.

In recent years, facial recognition has made significant progress in the field of anemia screening (see Table 1). Similar to our study, Mansour^19^ relied on an automatically adjustable frame to assist lip image acquisition. They constructed a binary classification model for anemia prediction with an accuracy of 99.28%. In contrast, our study emphasizes timeliness, non-intrusiveness, and convenience. Grounded in medical knowledge, we developed a DETR model focusing on the lip region. Without relying on additional tools, it not only effectively quantifies, visualizes, and replicates the diagnostic expertise of clinicians but also introduces a more granular three-category anemia classification. The time required for inference and analysis is significantly shorter than that of traditional hematology analyzers (usually 3–15 min). The model achieved an average accuracy of 85%, with higher precision in predicting moderate to severe anemia. Importantly, its direct compatibility with clinical decision-making significantly enhances its clinical value. Additionally, the model was trained on data collected in real clinical environments rather than controlled laboratory conditions, enhancing its external validity. Similar to our research philosophy, Hardalaç et al.^50^ developed a neonatal jaundice severity recognition model based on the characteristics of bilirubin-dependent skin color changes. Utilizing smart imaging devices, they captured images of multiple regions, such as the head and arms. Their study confirmed that non-invasive visual biomarkers can be safely utilized in clinical practice, which is highly encouraging to us. Likewise, the anemia detection model developed in this study—grounded in the absorption spectral characteristics of iron ions in hemoglobin and relying on the lip region—demonstrates significant potential for clinical deployment. It holds promise for integration into smartphone platforms to further improve the practicality of anemia screening in real-world environments. Meanwhile, this study explored the possibility of using local facial regions or a combination of regions to replace full-face images, aiming to eliminate privacy concerns. Although reliable privacy protection strategies have been implemented in data transmission and processing, subjects often remain concerned about privacy regarding facial recognition technology. Focusing on lip region features, our model achieved performance superior to that of full-face recognition models. We believe that relying on multiple anatomical regions highly correlated with anemia severity, such as the lips and cheeks, holds promise for achieving even better classification performance. Therefore, by only improving facial video acquisition methods, it is possible to avoid capturing full-face information at the source, thereby achieving better privacy protection. The incorporation of visual interpretation not only enhances model transparency but also demonstrates alignment with established clinical knowledge and findings documented in prior research. This partially alleviates the challenges posed by the “black box” nature of deep learning models^51^, thereby enhancing their potential for wider adoption and practical implementation. More importantly, we have endeavored to construct a “responsible” model designed to support high-stakes clinical decision-making. A key feature of this model is its ability to abstain from making a prediction when it lacks confidence. This “rejection option” prevents the model from being compelled to commit to a potentially erroneous classification, thereby mitigating the substantial risks associated with misdiagnosis in critical scenarios.

This study utilized deep learning techniques to demonstrate that lip region images are effective predictors of hemoglobin levels. The profound implication of this finding is that facial imaging offers a viable, non-invasive modality to assess disease status, either as a standalone tool or in synergy with traditional biomarkers. Central to our approach is that the model effectively quantifies, visualizes, and replicates the diagnostic expertise of clinicians. This innovation in disease detection is not restricted by the operator’s seniority or medical background, and it simultaneously ensures high accuracy and timeliness. In fact, whether in emergency rescue scenarios or home-based diagnostic and treatment settings, the most urgent priority is to accurately assess the severity of the condition within the target population. This capability enables prompt patient stratification and targeted treatment, ultimately saving more lives. For instance, consider a patient with a fall injury from a height and a preserved level of consciousness admitted to the emergency department. A junior physician attends to the case and suspects occult bleeding. Using our model at the bedside, the physician rapidly identifies the anemia severity as Category II. If a retest 5 min later, while awaiting laboratory results, shows progression to Category III, the patient should be immediately classified as high risk. This requires mobilizing additional medical resources and coordinating blood transfusion or surgical evaluation. If the model cannot yield a conclusion, the patient should still be closely monitored. Vital signs must be observed, and laboratory results tracked to prevent missed diagnosis. Consequently, the anemia detection model developed in this study is suitable for rapid dynamic assessment in emergency settings. It is also applicable for population screening in resource-limited areas. Furthermore, it supports long-term monitoring and early warning for community residents. By assisting treatment decisions and resource allocation, this model demonstrates significant potential for future application. Building upon the “hibernation” hypothesis, Madrigal-Garcia et al.^52^ provided the first systematic evidence linking facial action units (AUs) to patient prognosis. Our research extends this line of inquiry by establishing a direct mapping between specific facial regions and Hb levels, thereby laying a foundational basis for using facial features in the evaluation of conditions like anemia or patient prognosis with traumatic hemorrhagic bleeding. Despite the widespread implementation of sophisticated privacy protection strategies across data management, transmission, and processing, public concerns regarding privacy and security remain a significant challenge in the context of facial recognition technology deployment. This study introduces a detection model concentrating on features exclusively from the lip region, exploring a novel approach to enhancing privacy safeguards. Drawing on the experimental results, we suggest that a hybrid model integrating lip features with those from other anemia-correlated regions, such as the cheeks, holds the potential to significantly improve classification accuracy. Consequently, should this approach prove effective, modifying acquisition protocols to emphasize non-identifiable local features that are rich in disease information could obviate the need for full facial data collection at the source, thereby strengthening privacy without compromising analytical performance.

Our study still has several limitations. The sample size is relatively small, and the cohort consists entirely of Asians. We did not include multi-ethnic populations with different skin tones, which limits the generalizability of the current model. It is well established that genetics determines constitutive skin color. This intrinsic color is reinforced by facultative melanogenesis and tanning reactions^53^. Increased sun exposure and older age lead to greater pigmentation^54,55^, which significantly affects lip color. Therefore, the primary factors constraining model performance in this study may be the wide age range of the participants (18 to 95 years) and the lack of consideration for altitude of residence. This persists despite our efforts to expand the sample size to account for individual heterogeneity. Additionally, the data were collected from May to August in the same indoor environment with suitable temperature and humidity. While this eliminated the influence of environmental factors on lip moisture, we did not account for the severity of the patients’ conditions or their food and water intake. Consequently, we cannot rule out interference from internal factors affecting lip dryness, which could lead to misjudgment. However, the specific mechanism by which lip dryness affects model predictions remains unclear. Nevertheless, the successful application of multi-modal data fusion in enhancing AI model performance^56–58^ suggests that integrating the aforementioned information with facial images holds promise for further improvement. In the next steps, we will continue to expand the sample size. We plan to conduct multi-center and multi-ethnic validation along with stratified studies. Furthermore, we will explore privacy-preserving anemia detection models that fuse multiple facial regions, such as the lips and cheeks.

## Conclusions

The DETR model, concentrating on lip region image recognition, emerges as a promising and reliable tool for anemia severity assessment. By integrating high diagnostic accuracy, rapid processing, broad accessibility, user-friendliness, and medical interpretability, the model confers significant clinical utility and practical applicability. The model is expected to be deployed across diverse clinical and non-clinical settings, including emergency triage, large-scale population screening, and home-based health monitoring.

## Materials and methods

### Research design

In this prospective observational study, we developed and validated a deep learning model to assess the severity of anemia using facial video data collected from emergency department patients. The data processing pipeline encompassed frame extraction, quality screening, stratification by Hb values, manual annotation of lip and full-face regions, and data partitioning. The model was trained and evaluated on this dataset and subsequently benchmarked against diagnostic assessments provided by physicians with varying levels of clinical expertise, in order to evaluate its performance.

### Data collection

This study included patients who were treated at the Emergency Department of the First Medical Center, Chinese People’s Liberation Army (PLA) General Hospital, between May 2025 and August 2025. Inclusion criteria were: (a) age ≥ 18 years; (b) having undergone a complete routine blood test (complete blood count, CBC); (c) provision of written informed consent. Exclusion criteria were: (a) severe facial obstruction due to endotracheal intubation or oxygen mask therapy; (b) the presence of non-hemorrhagic conditions affecting facial coloration (e.g., jaundice, carbon monoxide poisoning, or hypoxemia, ect.); (c) interference on the face from makeup, bloodstains, dirt, or other contaminants. This study was approved by the Ethics Committee of the PLA General Hospital (Approval No. S2025-264-01). All methods were performed in accordance with the relevant guidelines and regulations. All subjects and/or their legal guardians have signed the informed consent form. Consent for publication: Written informed consent for publication of the unrecognizable information and/or images in this article was obtained from all participants (and/or their legal guardians). Furthermore, we will strictly abide by the principles of privacy protection and ensure the confidentiality of the subjects’ data.

Video acquisition was performed under the following standardized conditions: (a) Environment: Stable ambient lighting and temperature were maintained in the emergency room. (b) Timing: The video was captured within 30 min before or after the patient’s routine blood test (complete blood count, CBC). (c) Patient State: The patient was positioned supine and instructed to remain still to minimize facial occlusion and emotional interference. (d) Equipment and Settings: An AIM75-WIFI Pad ^16^ was used with the following settings: 1080p resolution, 30 fps frame rate, Auto mode, and autofocus enabled. (e) Technical Requirements: The device was positioned approximately 40 cm vertically from the patient’s face to ensure the entire face was in the frame. The recording duration was 20–30 s. The left and right deviation angles of the Pad were limited to 15°.

To ensure data integrity, the collection process should adhere to the following standards. First, direct sunlight must be avoided to prevent excessive contrast between highlights and shadows on the face. Second, underexposure caused by backlit environments must be minimized. Third, adequate and evenly distributed indoor lighting is essential to maintain image clarity and an optimal signal-to-noise ratio. Finally, all collection activities must be performed solely by clinical physicians with at least two years of clinical experience and who have completed at least one hour of in-person training prior to independent task execution.

Following collection, videos were stored in MP4 format, while clinical data—including demographics, major diagnostic categories, and Hb levels—was systematically retrieved from the Hospital Information System (HIS) and outpatient or emergency systems and compiled into Excel for analysis. Furthermore, comprehensive data de-identification measures were implemented, including replacing direct identifiers such as names and IDs with unique numerical codes, ensuring local deployment with document-level encryption, and enforcing stringent access controls to restrict original data access solely to authorized personnel.

### Data preprocessing and annotation

#### Image acquisition and quality control

Based on previous image acquisition strategies ^59^, one frame was extracted at a fixed interval of 100 frames from each video. For samples from underrepresented categories, an additional frame was extracted every 30 frames to mitigate class imbalance. All extracted facial images were then assessed for quality by two trained evaluators, who systematically excluded any image of poor quality due to blurring, uneven illumination, excessive noise, or motion artifacts.

#### Annotation of both lip and full-face regions

Lip region annotation for the partitioned training and testing cohort was performed by two researchers specializing in facial recognition using the LabelImg tool ^60^, to facilitate focused analysis of the lip region. Based on medical knowledge, the lip ROI was rigorously defined as a rectangular area encompassing the vermilion of the lip and its adjacent tissue, extending 3–5 mm beyond the mouth corners and the outer contours of the upper and lower lips, to preserve sufficient contextual information relevant to clinical evaluation. Simultaneously, LabelImg was used to annotate the entire facial region—from the forehead to the mandible and laterally to the inner contours of both earlobes—to facilitate holistic analysis of full-face images.

#### Data classification and partitioning

Face images were labeled into three categories based on hemoglobin values from routine blood tests. Category I was defined as HB > 9 g/dL, Category II as 9 g/dL ≥ HB ≥ 6 g/dL, and Category III as HB < 6 g/dL. This provided the model with labeled data containing known correct classes to learn how to map features to specific categories. Following a patient-level splitting principle, the face image dataset was randomly divided into a training set and a test set at a ratio of 8:2. Multiple frames derived from the same patient were assigned to the same set. This practice strictly guaranteed the independence of patient-level data.

#### Data augmentation approach

To address the challenge of model overfitting on a limited dataset, a multi-level data augmentation strategy was employed. This strategy first incorporated random horizontal flipping (p = 0.5) to bolster robustness against variations in facial orientation. A dual-component multi-scale approach was then applied. The first component, single-step scaling, randomly resized images using one of 11 predefined scales, with widths ranging from 480 to 800 pixels at a fixed height of 1333 pixels while maintaining the original aspect ratio. The second component utilized a three-stage pipeline: (a) initial magnification via random upscaling to one of three large dimensions (width: 400/500/600px; height: 4200px); (b) random cropping to extract a 384 × 600 pixel region of interest; (c) Scale standardization was identical to Strategy 1. One of 11 preset scales was randomly selected for final adjustment. Collectively, these augmentation techniques substantially enhance the training data’s diversity, improving the model’s capacity to accurately recognize lip and full-facial features under diverse scales, orientations, and lighting conditions.

### Model development

**Fig. 4:**
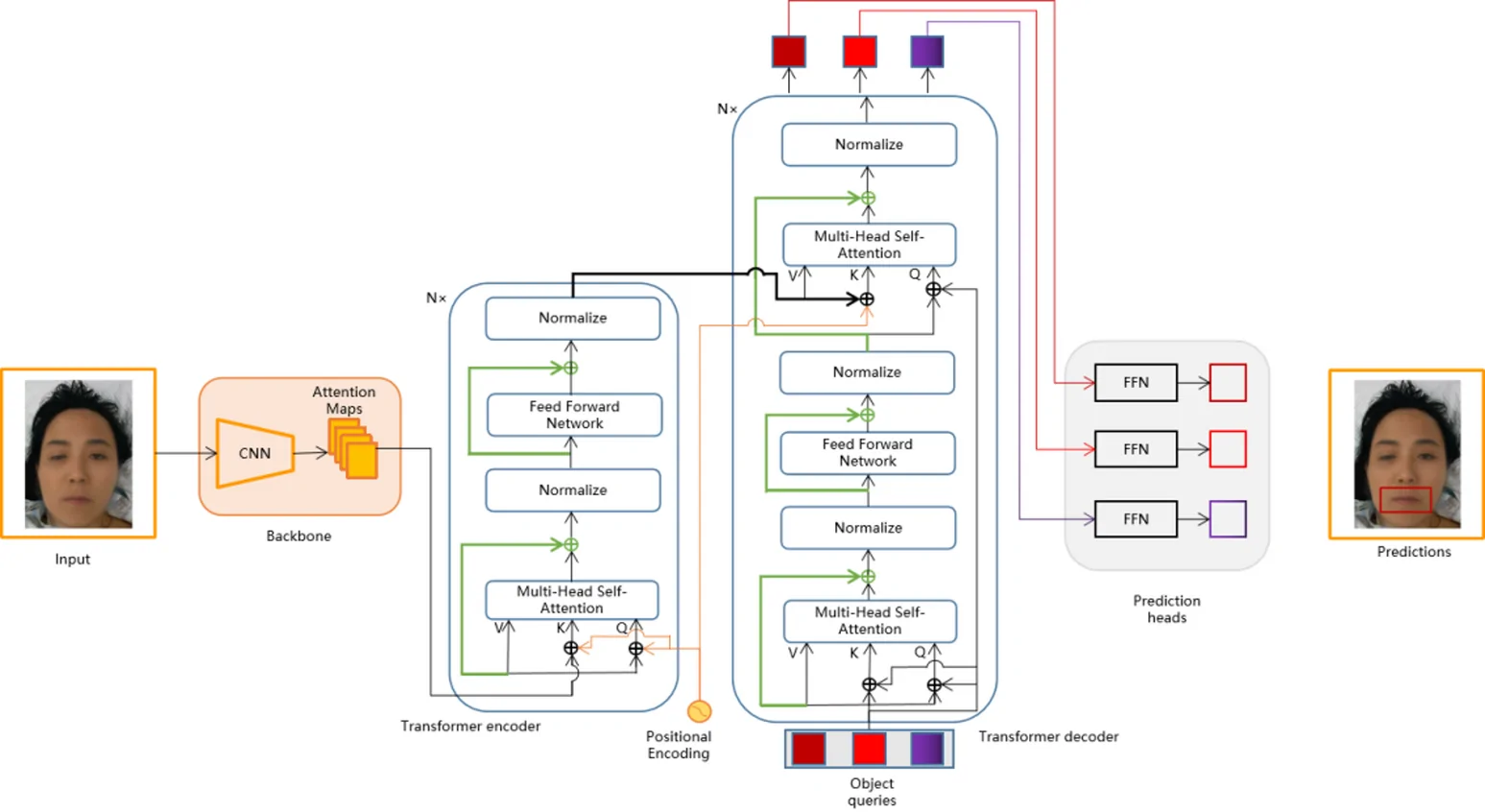
Framework diagram for model development.

Employing the same data partitioning strategy, Model A was developed to evaluate anemia severity using lip-annotated images as input. In contrast, Model B was developed using full-face annotated images as a comparative baseline. To ensure a fair and valid comparison, both models utilize an identical network architecture (Fig. 4).

#### The extraction of region of interest (ROI)

**Fig. 5:**
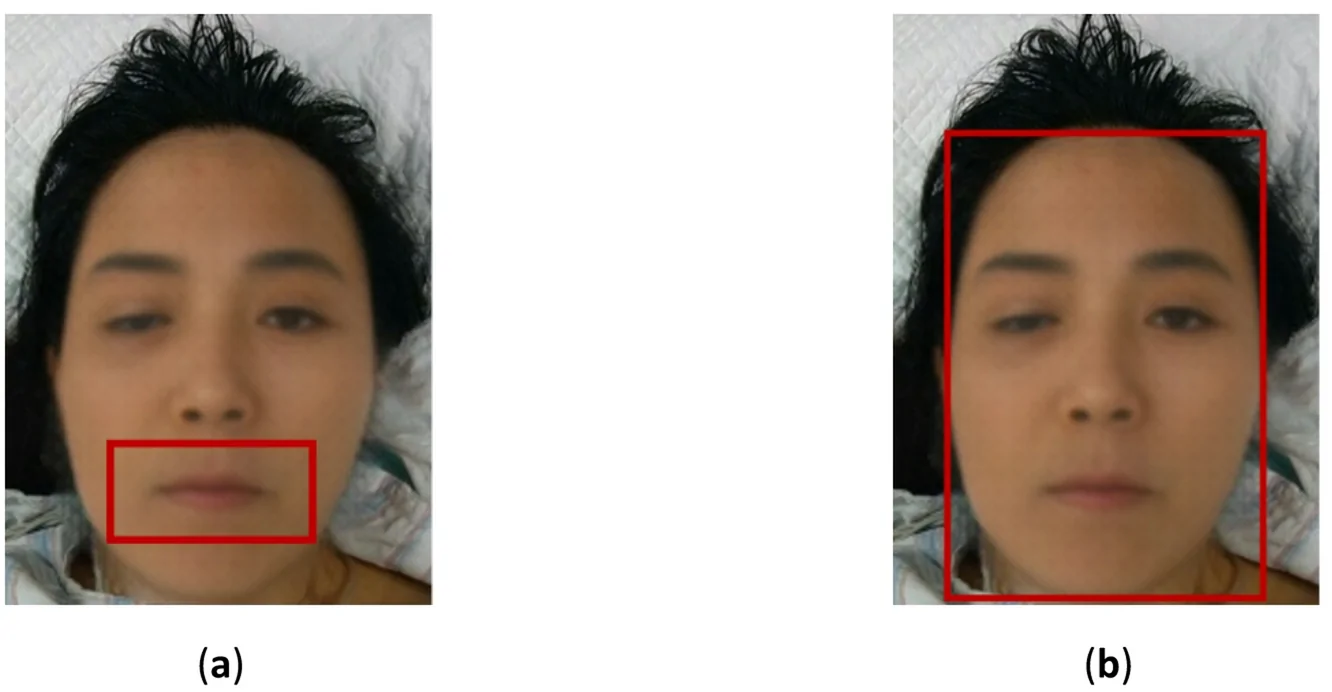
The ROI for Each Model. (**a**) the ROI for Model A; (**b**) the ROI for Model B.

The lip region, annotated with LabelImg, was defined as the ROI for Model A, while the entire facial area was used as the ROI for Model B, as shown in Fig. 5.

#### Feature extraction architecture

The feature extraction process was implemented using the DETR architecture, a process comprising three key stages: (a) extracting initial features through the ResNet-50 backbone network; (b) enhancing these features via Transformer encoders to capture color, texture, and morphological characteristics; (c) employing the Transformer decoder to refine learnable query embeddings, ultimately generating the final feature representations for object detection.

#### Model training

We conducted model training on an NVIDIA RTX 3090 GPU, utilizing PyTorch 2.5.1 as the deep learning platform and implementing the DETR model within the mmdetection 3.3.0 framework.

The training parameters were configured as follows: a batch size of 2, the AdamW optimizer, an initial learning rate of 1 × 10⁻^4^, a weight decay rate of 1 × 10⁻^4^, and a total of 30 epochs. Detection performance was optimized using a composite loss function, which integrates three distinct components via the Hungarian Assigner to determine the optimal prediction-to-ground-truth assignment. Specifically, the classification loss employs Focal Loss (weight: 2.0; α = 0.25, γ = 2.0) to address class imbalance. For bounding box regression, L1 Loss (weight: 5.0) minimizes coordinate errors, while GIoU Loss (weight: 2.0) refines the spatial alignment between predicted and ground-truth boxes. This multi-faceted loss strategy ensures comprehensive optimization by simultaneously mitigating class imbalance, refining coordinate accuracy, and enhancing overall shape alignment.

#### Model evaluation

The model’s performance was systematically assessed on the testing cohort. For each sample, the predictions and ground-truth labels were recorded to calculate a comprehensive set of metrics. These metrics included mean average precision (mAP), average accuracy, average recall, average inference time, and the confusion matrix, among other relevant indicators. For the mAP evaluation, an Intersection over Union (IoU) threshold of 0.5 was used. This choice was aligned with the clinical requirements of anemia detection, where the primary goal is to reliably detect symptoms rather than achieve pixel-perfect localization. Consequently, minor positional deviations are deemed acceptable, as a 50% overlap is sufficient to confirm localization of the relevant lip or facial region.

### Physicians interpretation and comparison

The study recruited four physicians: two senior emergency physicians with > 8 years of experience and two junior physicians with < 3 years of experience. Each participant independently and sequentially evaluated each sample in the test cohort using their clinical judgment. For each sample, one image was randomly selected for evaluation. The experiment was conducted in a controlled indoor environment to minimize external interference, and all communication between participants was prohibited throughout the process. Images were displayed sequentially on a laptop (Model: Honor MagicBook X 16 Pro; Resolution: 1920 × 1080 pixels). Two research assistants were present to supervise the session and document the duration of each evaluation. Classification was performed using the following Hb thresholds: Category I (Hb > 9 g/dL), Category II (6 g/dL ≤ Hb ≤ 9 g/dL), and Category III (Hb < 6 g/dL). The diagnostic performance of each physician was assessed with two primary metrics: average accuracy and average evaluation time. Meanwhile, Model A was used to re-evaluate the images used for physician interpretation. To determine whether the sample size was sufficient to detect significant differences between Model A and the physicians, a McNemar test was performed. This test was based on the results of Model A and the assessment results of the most accurate physician.

### Statistical analysis methods

Statistical analyses were performed using SPSS 27.0, and data visualizations were generated in Python. The normality of continuous variables was assessed. Normally distributed data were expressed as mean ± standard deviation (SD) and compared using the independent samples t-test. Data with skewed distributions were reported as median (Interquartile Range, IQR: P25, P75) and compared using the Mann–Whitney U test. Categorical data were expressed as percentages (%). Comparisons of rates were conducted using the Chi-square(χ^2^) test. Fisher’s exact test was applied when the expected frequency was less than 5. Statistical significance was defined as *P* < 0.05.

## Supplementary Information

Below is the link to the electronic supplementary material.

Supplementary Material

## Data Availability

The data that support the findings of this study are not publicly available due to privacy and ethical restrictions. Data are available from the corresponding author, Lili Wang, upon reasonable request and after approval from the Institutional Review Board (IRB) of Your Institution.
