# Supplementary material for: Development of an anemia detection model in emergency departments using lip region images based on medical knowledge and deep learning technology
Source: Sci Rep. 2026 Jun 20;16:28255. doi: 10.1038/s41598-026-40210-5 (PMC13558718; doi:10.1038/s41598-026-40210-5)
Supplement: Supplementary file 1 [file 41598_2026_40210_MOESM1_ESM.docx]

Supplementary materials 1

**Table 1.** Baseline Characteristics of the Training Cohort and the Test Cohort

| **Characteristics** | **Training cohort** | **Test cohort** | **P value** |
| --- | --- | --- | --- |
| **Category,n(%)** |  |  | 0.223 |
| Category I | 73(30.2) | 26(34.7) |  |
| Category II | 144(59.5) | 46(61.3) |  |
| Category III | 25(10.3) | 3(4.0) |  |
| Age(yd) | 67.5(54,77) | 62.79±16.422 | 0.095 |
| **Gender,n(%)** |  |  | 0.927 |
| Male | 147(60.7) | 46(61.3) |  |
| Female | 95(39.3) | 29(38.7) |  |
| Hemoglobin | 79(70,99.5) | 84(73,106) | 0.076 |
| **Major Diseases,n(%)** |  |  | 0.026 |
| Digestive System | 93(38.4) | 27(37.3) |  |
| Circulatory System | 60(24.8) | 16(21.3) |  |
| Respiratory System | 26(10.7) | 11(14.7) |  |
| Urinary System | 16(6.6) | 4(5.3) |  |
| Nervous System | 19(7.9) | 1(1.3) |  |
| Musculoskeletal System | 14(5.8) | 3(4.0) |  |
| Hematologic System | 10(4.1) | 5(6.7) |  |
| Other Body Systems | 4(1.7) | 7(9.3) |  |

Supplementary materials 2

**Table 1.** List of Model A and Physician Assessment Results

| **Number** | **Hb Level**  **(g/dL)** | **Anemia Category** | **Evaluation result** | | | | |
| --- | --- | --- | --- | --- | --- | --- | --- |
|  |  |  | **Model A** | **Senior Physician 1** | **Senior Physician 2** | **Junior Physician 1** | **Junior Physician 2** |
| 1 | 151 | Ⅰ | Ⅰ | Ⅰ | Ⅱ | Ⅰ | Ⅰ |
| 2 | 73 | Ⅱ | Ⅱ | Ⅱ | Ⅱ | Ⅰ | Ⅱ |
| 3 | 85 | Ⅱ | Ⅱ | Ⅱ | Ⅱ | Ⅱ | Ⅱ |
| 4 | 73 | Ⅱ | Ⅱ | Ⅰ | Ⅱ | Ⅱ | Ⅱ |
| 5 | 82 | Ⅱ | Ⅱ | Ⅱ | Ⅰ | Ⅱ | Ⅱ |
| 6 | 67 | Ⅱ | Ⅱ | Ⅰ | Ⅰ | Ⅱ | Ⅱ |
| 7 | 104 | Ⅰ | Ⅲ | Ⅱ | Ⅰ | Ⅰ | Ⅰ |
| 8 | 80 | Ⅱ | Ⅱ | Ⅱ | Ⅱ | Ⅲ | Ⅱ |
| 9 | 98 | Ⅰ | Ⅱ | Ⅰ | Ⅱ | Ⅱ | Ⅱ |
| 10 | 89 | Ⅱ | Ⅱ | Ⅰ | Ⅲ | Ⅱ | Ⅱ |
| 11 | 81 | Ⅱ | Ⅱ | Ⅰ | Ⅲ | Ⅲ | Ⅲ |
| 12 | 67 | Ⅱ | Ⅱ | Ⅱ | Ⅲ | Ⅲ | Ⅲ |
| 13 | 98 | Ⅰ | Ⅰ | Ⅰ | Ⅰ | Ⅱ | Ⅱ |
| 14 | 102 | Ⅰ | Ⅰ | Ⅱ | Ⅰ | Ⅱ | Ⅱ |
| 15 | 88 | Ⅱ | Ⅱ | Ⅲ | Ⅰ | Ⅱ | Ⅱ |
| 16 | 123 | Ⅰ | Ⅰ | Ⅰ | Ⅰ | Ⅱ | Ⅱ |
| 17 | 84 | Ⅱ | Ⅱ | Ⅱ | Ⅱ | Ⅲ | Ⅱ |
| 18 | 81 | Ⅱ | Ⅱ | Ⅱ | Ⅱ | Ⅲ | Ⅱ |
| 19 | 79 | Ⅱ | Ⅱ | Ⅱ | Ⅱ | Ⅱ | Ⅱ |
| 20 | 82 | Ⅱ | Ⅱ | Ⅲ | Ⅱ | Ⅰ | Ⅱ |
| 21 | 100 | Ⅰ | Ⅰ | Ⅱ | Ⅰ | Ⅰ | Ⅱ |
| 22 | 90 | Ⅱ | Ⅱ | Ⅱ | Ⅱ | Ⅲ | Ⅲ |
| 23 | 84 | Ⅱ | Ⅱ | Ⅰ | Ⅱ | Ⅱ | Ⅱ |
| 24 | 67 | Ⅱ | Ⅱ | Ⅱ | Ⅱ | Ⅱ | Ⅲ |
| 25 | 93 | Ⅰ | Ⅰ | Ⅰ | Ⅲ | Ⅰ | Ⅰ |
| 26 | 61 | Ⅱ | Ⅱ | Ⅱ | Ⅲ | Ⅲ | Ⅲ |
| 27 | 135 | Ⅰ | Ⅰ | Ⅰ | Ⅱ | Ⅲ | Ⅱ |
| 28 | 123 | Ⅰ | Ⅰ | Ⅱ | Ⅱ | Ⅲ | Ⅰ |
| 29 | 135 | Ⅰ | Ⅲ | Ⅱ | Ⅱ | Ⅲ | Ⅱ |
| 30 | 113 | Ⅰ | Ⅱ | Ⅰ | Ⅱ | Ⅱ | Ⅱ |
| 31 | 85 | Ⅱ | Ⅱ | Ⅰ | Ⅱ | Ⅰ | Ⅲ |
| 32 | 76 | Ⅱ | Ⅱ | Ⅲ | Ⅲ | Ⅲ | Ⅲ |
| 33 | 132 | Ⅰ | Ⅲ | Ⅰ | Ⅰ | Ⅰ | Ⅰ |
| 34 | 81 | Ⅱ | Ⅱ | Ⅱ | Ⅱ | Ⅱ | Ⅲ |
| 35 | 149 | Ⅰ | Ⅰ | Ⅰ | Ⅱ | Ⅰ | Ⅰ |
| 36 | 124 | Ⅰ | Ⅰ | Ⅰ | Ⅲ | Ⅰ | Ⅰ |
| 37 | 62 | Ⅱ | Ⅱ | Ⅱ | Ⅲ | Ⅱ | Ⅲ |
| 38 | 81 | Ⅱ | - | Ⅱ | Ⅱ | Ⅱ | Ⅲ |
| 39 | 83 | Ⅱ | Ⅱ | Ⅱ | Ⅱ | Ⅲ | Ⅱ |
| 40 | 81 | Ⅱ | Ⅱ | Ⅱ | Ⅰ | Ⅱ | Ⅲ |
| 41 | 64 | Ⅱ | Ⅱ | Ⅱ | Ⅲ | Ⅱ | Ⅲ |
| 42 | 69 | Ⅱ | Ⅱ | Ⅰ | Ⅰ | Ⅱ | Ⅱ |
| 43 | 73 | Ⅱ | Ⅱ | Ⅰ | Ⅱ | Ⅲ | Ⅱ |
| 44 | 114 | Ⅰ | Ⅰ | Ⅱ | Ⅰ | Ⅱ | Ⅰ |
| 45 | 69 | Ⅱ | Ⅱ | Ⅱ | Ⅱ | Ⅲ | Ⅱ |
| 46 | 80 | Ⅱ | Ⅱ | Ⅲ | Ⅱ | Ⅰ | Ⅱ |
| 47 | 90 | Ⅱ | Ⅱ | Ⅱ | Ⅱ | Ⅱ | Ⅱ |
| 48 | 80 | Ⅱ | Ⅲ | Ⅱ | Ⅱ | Ⅲ | Ⅱ |
| 49 | 46 | Ⅲ | Ⅲ | Ⅲ | Ⅲ | Ⅲ | Ⅲ |
| 50 | 84 | Ⅱ | Ⅱ | Ⅰ | Ⅱ | Ⅱ | Ⅱ |
| 51 | 84 | Ⅱ | Ⅱ | Ⅰ | Ⅱ | Ⅲ | Ⅲ |
| 52 | 81 | Ⅱ | Ⅱ | Ⅰ | Ⅲ | Ⅲ | Ⅲ |
| 53 | 72 | Ⅱ | Ⅲ | Ⅱ | Ⅲ | Ⅱ | Ⅲ |
| 54 | 75 | Ⅱ | Ⅱ | Ⅱ | Ⅱ | Ⅰ | Ⅱ |
| 55 | 89 | Ⅱ | Ⅱ | Ⅰ | Ⅱ | Ⅱ | Ⅲ |
| 56 | 73 | Ⅱ | Ⅱ | Ⅱ | Ⅱ | Ⅰ | Ⅱ |
| 57 | 67 | Ⅱ | Ⅱ | Ⅱ | Ⅰ | Ⅰ | Ⅲ |
| 58 | 59 | Ⅲ | Ⅱ | Ⅱ | Ⅱ | Ⅲ | Ⅱ |
| 59 | 140 | Ⅰ | Ⅰ | Ⅱ | Ⅰ | Ⅰ | Ⅱ |
| 60 | 75 | Ⅱ | Ⅱ | Ⅰ | Ⅱ | Ⅰ | Ⅱ |
| 61 | 112 | Ⅰ | Ⅰ | Ⅰ | Ⅰ | Ⅰ | Ⅱ |
| 62 | 144 | Ⅰ | Ⅰ | Ⅰ | Ⅰ | Ⅰ | Ⅲ |
| 63 | 74 | Ⅱ | Ⅱ | Ⅱ | Ⅱ | Ⅱ | Ⅱ |
| 64 | 111 | Ⅰ | Ⅱ | Ⅱ | Ⅱ | Ⅰ | Ⅱ |
| 65 | 42 | Ⅲ | Ⅲ | Ⅱ | Ⅱ | Ⅲ | Ⅲ |
| 66 | 136 | Ⅰ | Ⅱ | Ⅰ | Ⅰ | Ⅰ | Ⅱ |
| 67 | 130 | Ⅰ | Ⅰ | Ⅰ | Ⅰ | Ⅰ | Ⅱ |
| 68 | 97 | Ⅰ | Ⅰ | Ⅱ | Ⅱ | Ⅰ | Ⅱ |
| 69 | 88 | Ⅱ | Ⅱ | Ⅲ | Ⅱ | Ⅲ | Ⅱ |
| 70 | 106 | Ⅰ | Ⅰ | Ⅱ | Ⅰ | Ⅲ | Ⅰ |
| 71 | 64 | Ⅱ | Ⅱ | Ⅱ | Ⅱ | Ⅰ | Ⅱ |
| 72 | 155 | Ⅰ | Ⅲ | Ⅰ | Ⅰ | Ⅰ | Ⅱ |
| 73 | 65 | Ⅱ | Ⅱ | Ⅱ | Ⅱ | Ⅱ | Ⅲ |
| 74 | 165 | Ⅰ | Ⅰ | Ⅱ | Ⅰ | Ⅰ | Ⅱ |
| 75 | 78 | Ⅱ | Ⅱ | Ⅰ | Ⅲ | Ⅱ | Ⅲ |

**Table 2.** Contingency Table for McNemar’s Test Comparing Model A and Senior Physician 2

|  | **Senior Physician 2: Correct** | **Senior Physician 2: Incorrect** |
| --- | --- | --- |
| **Model A: Correct** | a=40  (Both Correct) | b =23  (Model Wins) |
| **Model A: Incorrect** | c=5  (Physician Wins) | d=6  (Both Incorrect) |
